# The Psychological Determinants of Obesity in Children and Adolescents

**DOI:** 10.34763/devperiodmed.20172103.208212

**Published:** 2017-10-28

**Authors:** Joanna Radoszewska

**Affiliations:** 1 Department of Child and Family Clinical Psychology, Child Clinical Psychology Laboratory, Faculty of Psychology, University of Warsaw, Warsaw Poland

**Keywords:** obesity, psychological mechanisms, emotional relationship with the mother, family relationships, body experience, body image, otyłość, mechanizmy psychicze, relacja emocjonalna z matką, relacje w rodzinie, przeżywanie ciała, obraz ciała

## Abstract

The aim of this article is to show selected psychological mechanisms involved in the onset and maintenance of obesity in children and youth.

This work presents a review of the literature related to the psychological determinants of obesity from different theoretical approaches. The role of the mother-child relationship, as well as the specific characteristics of the relationships within the family, have been emphasized in the onset of the disorder. Another topic discussed were the specifics of the body experience and certain body image distortions that promote the maintenance of the obese state. The control deficit caused by family relationships was also described.

## Streszczenie

Celem pracy jest przedstawienie wybranych mechanizmów psychicznych uwikłanych w powstawanie i utrzymywanie się otyłości u dzieci i osób w wieku dorastania. Zaprezentowano przegląd literatury psychologicznej dotyczącej psychicznych uwarunkowań otyłości w różnych ujęciach teoretycznych. Wskazano na szczególne znaczenie, jakie dla powstania zaburzenia może mieć relacja emocjonalna, w jakiej dziecko pozostaje z matką, oraz właściwości relacji w jego rodzinie. Omówiono specyfikę doświadczania ciała oraz specyficzne zaburzenie obrazu ciała sprzyjające utrzymywaniu się otyłości. Opisano deficyt w sprawowaniu kontroli będący skutkiem relacji w rodzinie.

Clinical psychologists view obesity as a disorder due to the scale of the problem for the person that is affected. Obesity becomes a clinical problem when it negatively influences the quality of the person’s psychological, interpersonal, and somatic functioning [1]. In a situation when the patient is a child, it is the child’s parents who seek specialist help due to the difficulties that the child has exhibited. People who are obese are sent to psychologists as a result of their emotional difficulties, lack of success in social functioning, and health problems [2].

Thinking about obesity as a disorder comes from the existence of certain objective anomalies. In the case of obesity, the problems are inappropriate (excessive) eating and inappropriate (excessive) body mass. It is commonly accepted that such behaviour (the compulsive consumption of food), as well as somatic functioning (excessive body weight) are signs of difficulties in a person’s psychological functioning. Based on scientific studies and the data gathered from clinical practice, psychologists describe the specifics of the psychological difficulties of these obese people [1].

The empirical and clinical data presented in this article comes from observation and examination of children and their families, for whom obesity is considered to be a problem. It is viewed as such, since the help of specialists (doctors, dieticians, psychologists) was sought by these people. The problems of “the families and the children” discussed in this article involve the group receiving obesity treatment. In other words, it does not involve all obese children and their families.

The psychological mechanisms behind the onset and maintenance of obesity are the object of inquiry in scientific studies for psychologists with different theoretical backgrounds. What is particularly emphasized, is the emotional relationship between the mother and the child, as well as the relationship of the child with their parents.

In terms of the onset and maintenance of obesity from a psychoanalytical approach, psychologists concentrate on the role of the compulsive consumption of food in the context of child development. Obesity in children can be seen as an effect of a troubled mother-child relationship [3]. It can be difficult for the mother of an obese child to give meaning to the child’s behaviour. She can be unable to differentiate between the child’s emotional expressions and biological needs. For instance, prematurely born children with very low birth weight, or children with genetic disorders often send weak signals that are difficult to differentiate and are often mutually exclusive. Psychologists point to the existent deficits in nonverbal and emotional communication in obese children and their mothers [4]. The mother’s adequate interpretation of the signals sent by her child, as well as the child’s responsiveness to outside stimuli are the basis for the correct development of the child, both in the somatic and the psychological sense. When the mother provides the child with food responding to the child’s behaviour that is indicative of hunger, children gradually develop the ability to distinguish between the need for food and their other needs [5]. When the emotional signals sent by the child are difficult for the mother to interpret, she can assume that the child is hungry, and thus she feeds him/ her. The lack of an adequate response to the child’s behaviour makes it impossible for the child to distinguish between various states - emotional ones - from hunger and satiety. The child does not learn how to identify and fulfill their somatic and psychological needs. Due to this unspecified internal arousal, the child searches for food in order to reduce their internal tension [1, 3, 6].

The disturbance in the relationship can manifest itself in the form of an overprotective attitude, ambivalence, and a rejection of the child by the mother. Mothers who are uncertain about their feelings and hostile towards their child can attempt to compensate by having an overprotective attitude, as well as with excessive feeding. Food becomes a substitute for love and safety. These mothers can also be overly concerned about the child’s physical safety and health. According to Hilda Bruch [3] it can lead to hypochondriac attitudes in children. Mothers of obese children can exhibit hostile attitudes towards the child’s peers, regarding them as threatening and undesirable. They experience their own children as unprepared for independent functioning with no parental supervision. These mothers can experience fear and insecurity in situations where they cannot control their child’s activities [3].

The feeding process can become the core element of the emotional exchange between the mother and the child. By giving food to the child who likes it and will eat a lot, the mother communicates love and at the same time protects herself from the feeling of maternal incompetence and from a sense of guilt. Moreover, the mother starts to associate love with gift-giving, feeding, and doing everything for the child. The limitations of food leads to the mother’s inability to give affection to her child.

According to Hilda Bruch, the difficulties in understanding the child described above can come from the mother’s problems. The mothers of obese children are more likely to experience depression and anxiety. This leads to an increase in self-concentration and, as a consequence, the inability to notice the signals that the child is sending. Bruch emphasizes the emotional aspect in the onset of obesity. She points to the specifics of the relationships in the family with an obese child. She describes the mothers of obese children as women who experience the feeling of loneliness and abandonment in their relationship with their partner, who are objectively alone, and are unable to handle their own situation. She also talks about mothers who are competitive and who feel constantly judged by their partners. An obese child is to be there to support the mother, allow her to be positively perceived by others, and not to distract her while she is achieving her life goals [3].

The mothers of children who receive obesity treatment also recall a specific way of experiencing their early relationship with their child. The mothers of obese adolescents were more likely to mention traumatic events in the early childhood of their children than the mothers of children who had a healthy weight. These traumatic events consisted of the following: 1. Threats to the perinatal life of the child, 2. Hospitalisation of the child in the first year of life, 3. The child’s refusal to eat, 4. The occurrence of a somatic disease with a severe course, 5. Others (for example a physical injury- he fell out of the baby carriage, she bumped her head) [7]. Giving food to a child gains a special meaning, especially for the mother - it can be seen by her as a confirmation of her maternal competence, while the child’s acceptance of the food and weight gain is the sign of the child’s health and will to live. It would appear that the maternal function described by Stern regarding keeping the child alive and providing them with optimal conditions for development seems to be especially significant in terms of the mothers who were examined in this study. It relates to the child’s welfare activities, taking care of the somatic state, and the health of the child. The mother’s anxiety level can be increased by feeding difficulties and the child’s health problems [7].

Obesity can also be the result of the parent-parent and parents-children relationship. Using the psychosomatic family model, it can be assumed that the exhibition of this symptom (obesity) leads to the maintenance of the homeostasis of the family.

With this approach, obesity is viewed as the result of keeping the child in the early childhood dependency. This also hinders children from establishing their own identity and going through the separation process. The mother fulfills her emotional needs by treating the child as little and making him/ her dependent on her in terms of feeding. A troubled relationship between the parents can often lead to a child’s limited autonomy [8, 9].

In comparison to the families of people with a proper weight, families of obese children are often described as systems with limited autonomy. In these families, the boundaries between the family members are also difficult to distinguish. It is also more common that the family units of obese people seem to have a lower level of integrity, which often exhibits itself as a lack of a consistent and coherent parenting style. The parents of an obese child experience a strong bond with their own parents. In their marriage, the parents of obese children often have problems communicating emotions, especially aggression. The difficulty in separation is the consequence of a specific relationship between the parents and the obese child. What is often emphasized, is the existence of the child-parent dependency, which, can result in such difficulties as e.g. social functioning and peer relationships [3].

Richard Ganley [9] describes the families of obese people in a similar manner. Obese patients’ families can be characterised as overprotective, rigid, and unable to resolve conflicts. In comparison to the families with a member who is slightly overweight (no obesity treatment), families who have a member going through obesity treatment are described as more rigid, less effective in terms of communication between the spouses, having difficulty expressing anger, and negatively responding to the emotions of others [9].

W.J Doherty and J.E. Harkway (1990) describe the specific meaning of the weight of the child in a family, in which the parents are obese. “To be obese” means: to belong and be loyal to the family. From the child’s perspective, obesity can be seen as an element of the integrity with the parents. Any weight loss can be perceived as a sign of separation and a lack of loyalty towards the parents. For the child it means that he/ she is getting rid of the ways in which they identify with their mother and father. For the parents, a reduction in the child’s weight is also a loss. By losing the child, they experience the lack of a person who keeps the family together [8].

To sum up, obese people’s families to a large extent seem to resemble the families of people with anorexia or bulimia. They can be classified as centripetal families, with a tendency to hinder the child from experiencing autonomy in a relationship and in forming their own identity. In the same way as the families whose members experience eating disorders, they are characterised by: (1) a limited autonomy of each of the family members; (2) meeting the needs of most of the family members; (3) the pattern of dominance-submission between the parents; (4) the subordination of the individual’s needs and self-realization to family values and ideals. Similarly to families of people with bulimia, the specific role of food in the communication process can be seen [1, 10].

The difficulties in the mother-child relationship, as well as the specifics of their family functioning can result in a specific type of identity formation. There seems to be a relationship between the chosen characteristics of a person and the maintenance of obesity symptoms. Assuming that the origins of obesity can be found in psychosomatics, it can be said that this is a somatic indication of the coexistence of a person’s certain characteristics: submissive dependency, emotional frustration, a strong need for love, and a limited ability to deal with emotions. It is a physiological reflection of difficult emotional experiences-anxiety and depression [11].

It can be said that obesity is a manifestation of the failure of the functioning of a person’s psychological mechanisms.

Body experience is the core difficulty for obese people. Research scientists who follow the psychosomatic approach state that obese people do not give adequate names to their body experiences. Body sensations can be seen as non-specific signals informing them about internal arousal with neither somatic nor psychological characteristics. This makes it difficult for the person to distinguish emotions from hunger and satiety. Therefore, it is impossible for the obese person to adequately identify and fulfill their needs, both somatic and psychological. It can be said that obese people do not know or understand themselves.

The psychologists who investigate obesity all agree that body image is the core dimension that differentiates obese people from people with a healthy body mass. The difference is also noticeable in people who are getting treated for obesity and obese people who receive no help from specialists. People who gained weight after adolescence, experience body image distortions more often than those who have been obese since childhood. Body image distortions are also common in adolescents [12].

The term “body image” is most commonly defined as a person’s notion of the physical appearance and/ or their attitude towards it. Two elements tend to be emphasised: body perception and body concept [13].

Obese people tend to distort the size of their own body. These body image distortions most often manifest themselves in an under- or over-estimation of their body size. These distortions specifically exhibit themselves in an underestimation of the size of certain body parts. In the case of the body as whole, obese people, similarly to anorexia nervosa patients, tend to overestimate their size. These created distortions of the body image tend to be persistent; they remain in spite of weight loss [3, 14].

The second element of the body image and body concept is defined by psychologists as the person’s attitude towards their own body. Obese people are more often unhappy with their body, and they also tend to concentrate on their appearance. Due to the necessity of showing their appearance, obese people aim to avoid social situations. They often feel a lot of negative emotions towards their bodies. Obese people are much more unhappy with their bodies than slim people. According to a research scientist, negative bodyesteem is the internalised criticism of people in a person’s surroundings [14, 15].

There seems to be a specific body image distortion in obese people that promotes the maintenance of obesity.

The body image distortion exhibits itself in:

1. Excessive concentration on one’s appearance;
2. The belief that one’s appearance negatively influences the assessment of their achievements and activities;
3. Avoidance of social situations due to physical appearance
4. Excessive concentration on hiding their body or experiencing repulsiveness towards it [14].

This specific way of experiencing oneself manifests itself in the patient’s delusional belief that all they need to do is to make changes to their body in order to change the quality of their emotional, social, and cognitive sphere of functioning.

Kaplan and Kaplan [16] observed that obese people exhibit compulsive eating in situations that trigger anxiety, and that the consumption of food allows them to reduce that anxiety. They point to early learning mechanisms where the association between pleasure, safety (lack of anxiety), and feeding is learnt. They also described the difficulty of obese people in distinguishing between anxiety and hunger. The researchers believed that the source of this difficulty is the learned reaction of eating as a response to both hunger and anxiety [16, 17].

The loss of control over eating that characterises obese children, adolescents, and adults.

There seems to be a relationship between the loss of control and in experiencing negative emotional states, such as depression and anxiety. The loss of control over eating exhibits itself in compulsive consumption. The obese person can feel as though he or she is reducing any painful, negative emotions through the act of eating. Food is used as a regulator of their experienced emotions. The cognitive-behavioral approach introduced the term “emotional eating”, which is understood as a coping mechanism that allows a person to regulate and reduce negative emotional states. “Emotional eating” becomes a daily, indispensable way of coping, both with negative and positive emotions. The recognition, experiencing, and expression of these emotions can often be limited due to alexithymia 1Alexithymia: the lack of verbal ability to express one’s own inner experiences and the difficulty to identify their own emotional states (cf. Morosin, Riva, 1997 )., which is often present in obese people [18, 19, 20].

The ability to exercise control is shaped throughout the child’s developmental process. The control deficit can be determined by the parents’ relationship with the child: for example, overly controlling parents can lead to a child’s lack of control. Such children are unable to experience control; they do not develop abilities to control themselves or to influence the behaviours of others, as the control is exercised only by the parents. It seems that this disturbance in the balance between children’s self-control and their outside control (parents) is a typical “configuration of power” in the obese child’s family [1, 21].

It can be said that an obese person is a person who does not take into account his/ her internal signals and who has deficits in exercising control and in dealing with difficult emotions. Obesity seems to be the result of the child’s helplessness in a relationship with overly controlling parents.

Maintaining obesity in a child can also be promoted by the way that parents view the obesity. The parents of obese children are more likely to concentrate on the somatic characteristics of the child than the parents of children with a healthy weight. Obesity can also influence the quality of the parent-child relationship. Studies show that young, obese girls often face severe criticism from their parents, especially from their mothers. Parents, and most of the time mothers, perceive obesity as a source of the difficulty in their relationship. This can also prevent them from seeing other important problems [22].

## Conclusion

Obesity is a somatic symptom with a complex etiology. In the process of diagnosis and treatment, it is worth considering the psychological mechanisms involved in the onset and maintenance of obesity. Obesity can be significant in terms of the mother-child relationship and other relationships in the family. A child’s obesity can play a role in experiencing emotions and in social relationships with peers and adults [1]. One of the reasons for unsuccessful obesity treatment could be the exclusive concentration on the somatic aspect of the disorder.
